# Primary bone lymphoma: pictorial essay

**DOI:** 10.1590/0100-3984.2019.0137

**Published:** 2020

**Authors:** Flavio Scavone Stefanini, Franklin Wilson Caires Gois, Tiago Cesar Silva Borba de Arruda, Almir Galvão Vieira Bitencourt, Wagner Santana Cerqueira

**Affiliations:** 1 Department of Imaging, A.C.Camargo Cancer Center, São Paulo, SP, Brazil.

**Keywords:** Bone neoplasms/diagnostic imaging, Lymphoma/pathology, Radiology, Magnetic resonance imaging, Neoplasias ósseas/diagnóstico por imagem, Linfoma/patologia, Radiologia, Ressonância magnética

## Abstract

Primary bone lymphoma is a rare neoplasm that can initially present as local pain, a palpable mass, and pathologic fracture. It can also be discovered as an incidental finding on an imaging examination. It is defined as a bone marrow tumor with no involvement of other sites, lasting at least six months. The diagnosis is confirmed by biopsy and immunohistochemical analysis. Although the imaging characteristics are nonspecific, there are certain findings that, when correlated with clinical and epidemiological aspects, can increase the level of suspicion of primary bone lymphoma. The classic imaging aspect is a bone lesion with a soft-tissue component that preserves the cortical layer more than would be expected given the invasive nature of the lesion. Magnetic resonance imaging is the best imaging method to evaluate the extent of involvement of adjacent compartments, whereas computed tomography depicts the cortical layer in greater detail, as well as being an important tool for biopsy guidance. Other imaging modalities are also discussed, such as X-ray, ultrasound, bone scintigraphy, and positron emission tomography/computed tomography. The aim of this paper is to describe the most common findings obtained with the various imaging methods used in patients with a confirmed diagnosis of primary bone lymphoma.

## Abstract (pt)

Linfoma ósseo primário é uma neoplasia rara, apresentando-se com dor, massa palpável e fratura patológica ou, ainda, como achado incidental em exames de imagem. É definido por tumor medular ósseo com ausência de acometimento de outros sítios por pelo menos seis meses, sendo confirmado por biópsia e avaliação imuno-histoquímica. As características de imagem são inespecíficas, mas alguns achados, quando associados a outros aspectos clinicoepidemiológicos, podem ajudar e aumentar o nível de suspeição para esta entidade. Lesão óssea com componentes de partes moles e relativa preservação cortical maior que a esperada configura uma das principais características de linfoma ósseo primário. A ressonância magnética é o melhor método de imagem para avaliar a extensão da lesão óssea para os compartimentos de partes moles, enquanto a tomografia computadorizada avalia com detalhes a cortical óssea, sendo também uma ferramenta importante para orientação de biópsias. Outras modalidades de imagem também são discutidas, como radiografia, ultrassonografia, cintilografia óssea e tomografia por emissão de pósitrons/tomografia computadorizada. O objetivo deste trabalho é descrever os achados mais frequentes nos diferentes métodos de imagem utilizados em pacientes com linfoma ósseo primário com diagnóstico confirmado em um centro oncológico de referência.

## INTRODUCTION

Primary bone lymphoma (PBL) is a rare disease, accounting for only 3-7% of all primary bone neoplasms, approximately 5% of extranodal lymphomas, and less than 1% of all non-Hodgkin lymphomas^(1,2)^, diffuse large B-cell lymphoma being the most common^(1-4)^. They are extremely rare in the pediatric and young adult populations^(2)^.

The following criteria for the diagnosis of PBL are widely accepted^(1)^: the primary site of tumor origin being in the bone marrow, no other site indicating the existence of the lesion on physical or imaging examination; no lymphoma being identified at any other site within the first six months after the diagnosis of PBL; the diagnosis being confirmed by pathology and immunohistochemistry; a diagnosis of malignant lymphoma, with the exception of PBL and secondary bone lymphoma, having been excluded.

Lymphomatous involvement of bone occurs most often in the metadiaphysis^(1)^, the femur being the most affected site, followed by the tibia and humerus^(5)^, although there is considerable divergence in the literature between appendicular and axial involvement, especially in studies conducted in Japan^(3,6)^. Other sites of involvement include the pelvis, skull, and neck^(3,7)^. Multifocal lesions usually affect axial and appendicular bones^(5)^.

In most studies, PBL is diagnosed in patients between 50 and 70 years of age, primarily affecting males^(3)^. Clinically, PBL manifests as local pain, a palpable mass, or pathological fracture, and some patients develop B symptoms (fever, night sweats, and weight loss).

The clinical presentation and imaging findings of PBL, which will be discussed below, are nonspecific, with a wide range of possible differential diagnoses, including Ewing’s sarcoma, osteosarcoma, chondrosarcoma, metastases, and Langerhans cell histiocytosis^(2)^. However, the thorough evaluation of specific radiological patterns can increase the level of suspicion for PBL before tissue biopsy.

The objective of this pictorial essay is to illustrate the most frequent findings obtained with the various imaging methods used in patients with PBL.

## GENERAL IMAGING CHARACTERISTICS

Originating from the bone marrow, PBL is a neoplasm that has widely variable presentations on imaging, and the findings can overlap with those numerous non-neoplastic conditions, as well as with those of other benign or malignant neoplasms. One common, striking feature is a soft-tissue mass that is much larger than would be expected on the basis of the degree of cortical bone destruction. In other words, a mass that extends to soft tissues with relative preservation of the cortical bone may be typical of PBL^(4,7)^. It has been suggested that this pattern results from the spread of small, round, blue tumor cells throughout the cortical bone via Volkmann’s and Havers’ vascular canals to the surrounding soft tissues, rather than from a clear cortical break^(4,7)^.

## RADIOGRAPHY

**Figure 1:**
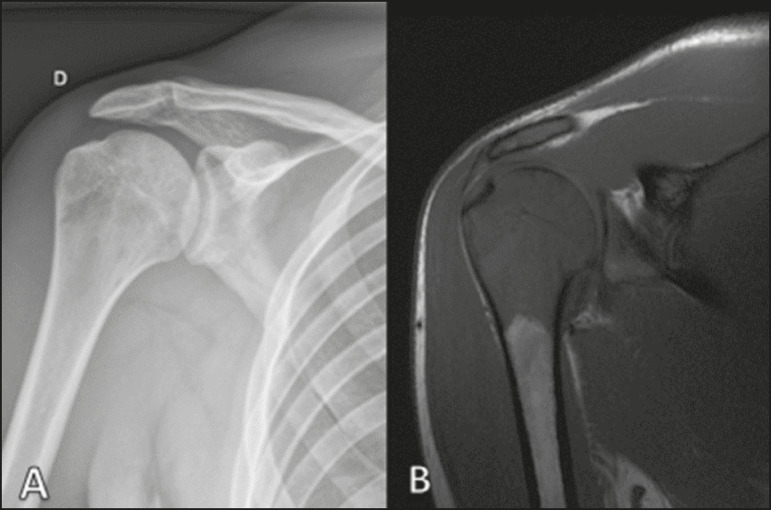
A 36-year-old male patient with spontaneous shoulder pain without improvement after treatment with analgesics. Anteroposterior X-ray (**A**) of the shoulder demonstrates texture change in the proximal metaepiphysis of the humerus. T1-weighted MRI sequence (**B**) shows bone marrow involvement of the metadiaphysis and humeral epiphysis.

Conventional X-ray is used in the initial assessment of the clinical complaint and can reveal aspects that merit further investigation. In this clinical context, the radiological appearance of a bone lymphoma can be normal or can include mild textural changes in the bone marrow^(1)^, as shown in Figure 1.

**Figure 2:**
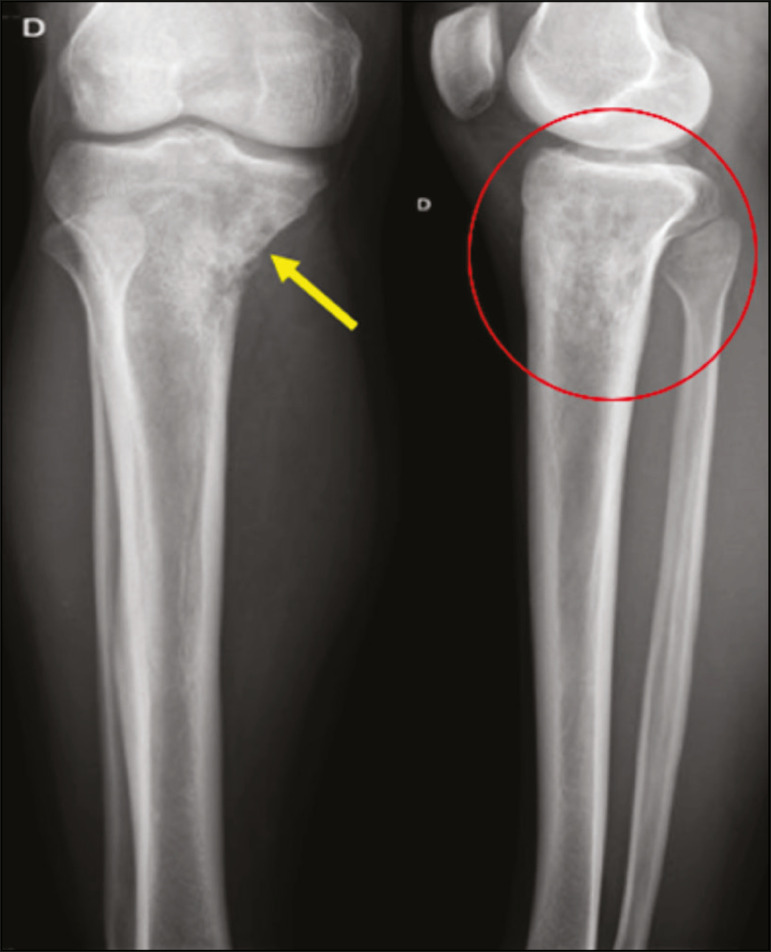
A 22-year-old male patient with intermittent pain in the right knee and progressive limitation of activities of daily living. Conventional X-rays of the leg (anteroposterior and lateral) showing a mixed, predominantly osteolytic, intramedullary lesion in the proximal metadiaphysis of the tibia, with a moth-eaten pattern and a wide zone of transition (circle), accompanied by slight irregularities and areas of cortical discontinuity (arrow).

Most reports of PBL describe an osteolytic pattern of infiltrative bone destruction with a wide transition zone or a moth-eaten appearance (Figure 2), and a periosteal reaction commonly reported to have a lamellar, multilamellar (“onion skin”), or discontinuous aspect^(1)^.

## ULTRASOUND

**Figure 3:**
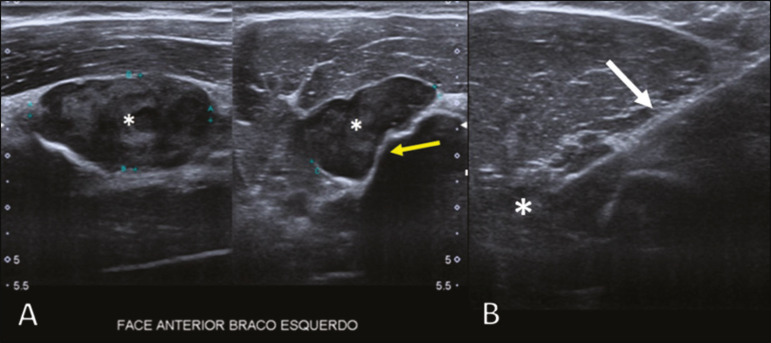
A 30-year-old male patient with pain in the left arm and B symptoms, submitted to biopsy of the soft tissue component after inconclusive bone biopsy. Ultrasound study delimits the dimensions of the soft tissue component (asterisks) and shows the path of the biopsy needle to the tumor component. The yellow arrow indicates the cortical bone related to the extraosseous tumor, and the white arrow indicates the biopsy needle.

Ultrasound has limited specificity to diagnose and differentiate PBL lesions. Its main function is to guide puncture or percutaneous biopsy of bone tumors that are accompanied by an extraosseous soft-tissue mass (Figure 3). Ultrasound can be used as an adjunct to magnetic resonance imaging (MRI) when secondary susceptibility artifacts prevent the assessment of specific areas^(8)^.

## COMPUTED TOMOGRAPHY

**Figure 4:**
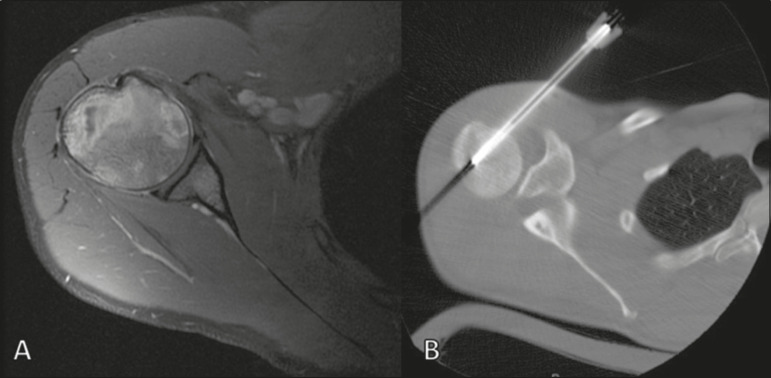
Same patient as Figure 1. Contrast-enhanced T1-weighted MRI examination (**A**) showing an infiltrative lesion in the humeral head. CT of the humerus (**B**) used in order to guide percutaneous biopsy.

Computed tomography (CT), in addition to being a widely used method to guide biopsies in cases of bone tumors (Figure 4), is an excellent method for delineating cortical destruction. Even if the other characteristics identified are nonspecific, the diagnosis of PBL should be considered whenever a large soft-tissue mass and abnormal bone marrow attenuation are observed without extensive cortical destruction proportional to the volume of the extraosseous lesion^(9)^.

## BONE SCINTIGRAPHY

Although radionuclide bone scintigraphy (^99m^Tc scintigraphy) is usually nonspecific, it helps to detect any active area in the skeleton, determining whether a tumor is latent or active^(7)^. Increased uptake of the tracer is seen in 98% of patients with PBL, that uptake (activity) being markedly increased in 64%^(7,10)^.

## POSITRON EMISSION TOMOGRAPHY/CT

**Figure 5:**
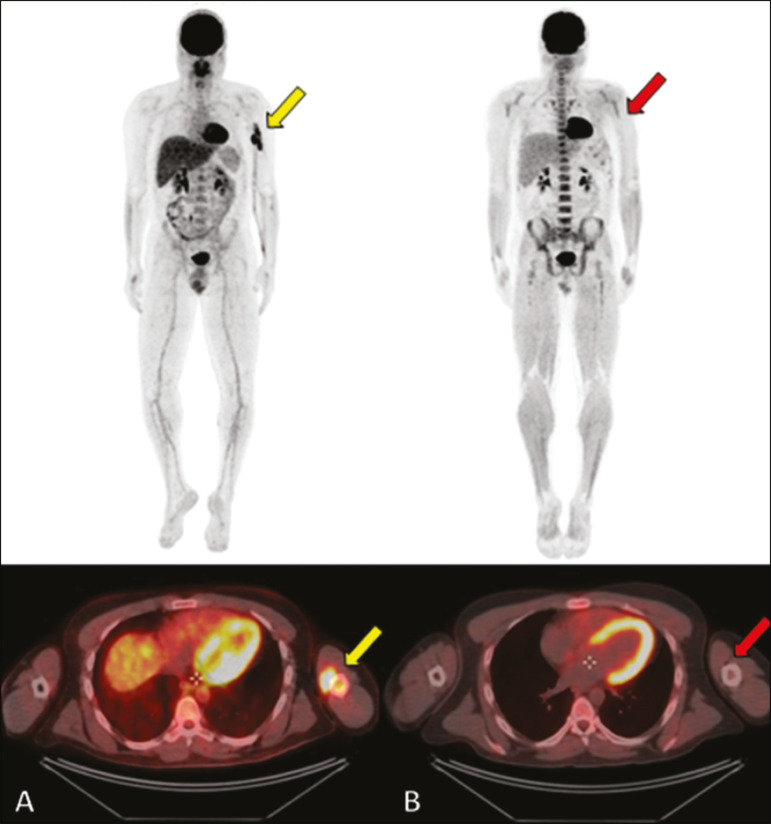
PET/CT examinations before and after chemotherapy (**A** and **B**, respectively) of the same patient depicted in Figure 3. Note the complete response after chemotherapy treatment, with no uptake of the radiopharmaceutical.

Positron emission tomography/CT (PET/CT) shows greater specificity and sensitivity than does conventional bone scintigraphy in the identification of lymphomatous infiltration of the skeleton^(1)^. In most cases, PBL presents as a hypermetabolic lesion on examination with fluorodeoxyglucose (Figure 5). In the follow-up of patients with PBL, PET/CT is an important tool to assess the presence of a viable lesion, being used even to define the therapeutic response. Post-therapeutic changes, such as tumor necrosis, granulation tissue, and fibrosis, are often difficult to distinguish from tumor remnants on CT and MRI. In this context, the presence or absence of uptake of the radiotracer in a PET/CT examination helps define more precisely the nature of the remaining tissue.

## MRI

Because it provides excellent soft-tissue contrast between compartments, MRI demonstrates well the relationship with neurovascular structures and shows the extent to which a lesion has invaded bone marrow, as well as the size and volume of the lesion. It allows tissue characterization by assessing signal intensities with different pulse sequences, the characteristics of contrast enhancement and the intensity of restricted diffusion on diffusion-weighted imaging^(4)^.

**Figure 6:**
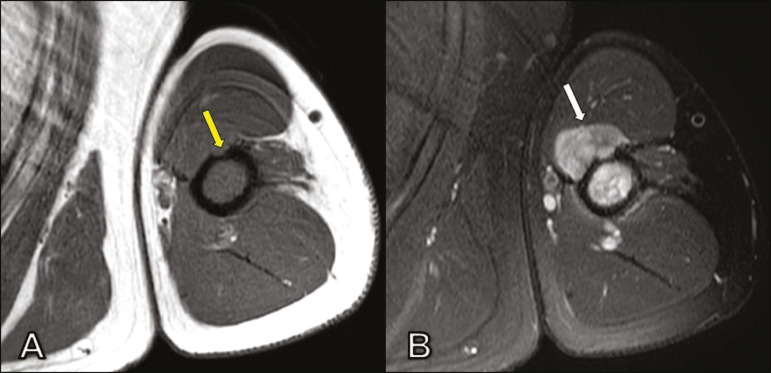
Same patient depicted in Figures 3 and 5. Axial T1-weighted MRI images before and after contrast administration (**A** and **B**, respectively), showing a defect in the anterior cortex (yellow arrow) communicating osseous lesion with soft tissue involvement (white arrow), indicating that the tumor has spread through the vascular/neurovascular canals.

**Figure 7:**
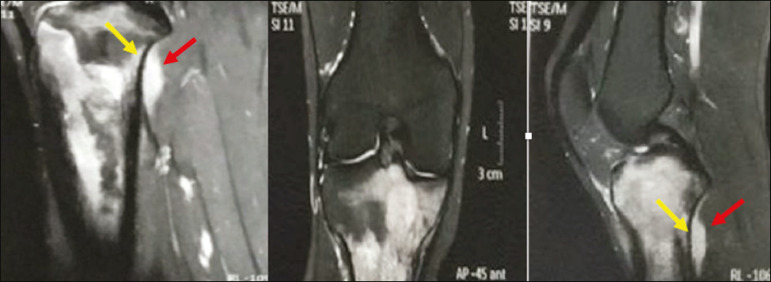
Same patient depicted in Figure 2. MRI (in different planes and slices) showing an infiltrative, ill-defined intramedullary bone lesion, located in the metadiaphysis of the proximal tibia, with low signal intensity on T1-weighted images, high signal intensity on T2-weighted images, and heterogeneous contrast enhancement. There is involvement of soft tissues in the posteromedial region (red arrow), with relatively well-preserved cortical bone in terms of the extent of extraosseous involvement (yellow arrow).

In patients with PBL, T1-weighted MRI sequences show areas of low signal intensity, and are generally the best sequences for revealing changes in bone marrow content. In contrast, those same areas show an intermediate signal on T2-weighted sequences (Figure 6). Peritumoral edema and reactive bone marrow changes can produce high signal intensity that varies depending on the degree of fibrosis^(1,4)^. Exhibiting varying degrees of contrast enhancement, PBLs, because of their relatively high cellularity, show restricted diffusion with a low apparent diffusion coefficient on diffusion-weighted sequences^(1)^. Finally, MRI is the best imaging method to assess the extent of bone injury in the soft-tissue compartments and the degree of cortical involvement in PBL, the cortical bone typically being preserved because of the infiltrative pattern of cortical penetration^(9)^, as depicted in Figure 7.

## CONCLUSION

Although PBL is a rare entity and its final diagnosis is carried out through pathological and immunohistochemical analysis, radiological evaluation is important for early diagnosis in incidental discoveries, biopsy guidance, staging, and evaluation of locoregional involvement, as well as follow-up and response assessment. Because there are no specific image characteristics, clinical, epidemiological, evolutionary and imaging knowledge helps guide the differential diagnosis of PBL, and for that we have an arsenal of imaging modalities that radiologists and general practitioners should be aware of, not only in terms of their interpretation but also in terms of the most appropriate indications for each modality.
